# Quality of life identification by unsupervised cluster analysis: A new approach to modelling the burden of endometriosis

**DOI:** 10.1371/journal.pone.0317178

**Published:** 2025-01-16

**Authors:** Alexandre Vallée, Maxence Arutkin, Pierre-François Ceccaldi, Jean-Marc Ayoubi

**Affiliations:** 1 Department of Epidemiology and Public Health, Foch hospital, Suresnes, France; 2 Département Universitaire de Santé Publique, Prévention, Observation, Territoires (SPOT), Université de Versailles, Saint-Quentin-en-Yvelines (UVSQ), Versailles, France; 3 School of Chemistry, Center for the Physics & Chemistry of Living Systems, Tel Aviv University, Tel Aviv, Israel; 4 Department of Obstetrics, Gynecology and Reproductive Medicine, Foch Hospital, Suresnes, France; 5 Medical School, University of Versailles, Saint-Quentin-en-Yvelines (UVSQ), Versailles, France; Dipartimento di Scienze Mediche e Chirugiche (DIMEC), Orsola Hospital, ITALY

## Abstract

**Background:**

Symptoms frequently associated with endometriosis affect quality of life (QoL). Our aim investigated the hypothesis that cluster analysis can be used to identify homogeneous phenotyping subgroups of women according to the burden of the endometriosis for their QoL, and then to investigate the phenotype differences observed between these subgroups.

**Methods:**

We developed an anonymous online survey, which received responses from 1,586 French women with endometriosis. K‐means, a major clustering algorithm, was performed to show structure in data and divide women into groups based on the burden of endometriosis. This was defined using 9 dimensions. Multivariable logistic regression was performed to highlight the association between QoL and several factors. Covariables were age, BMI, smoking, education, children, marital status and surgery.

**Results:**

K‐means clustering was implemented with 8 clusters (optimal CCC value of 17.2162). In one cluster, women presented a high level of QoL and represented 234 women for 60% of women with a high level of QoL, and another with 410 women for 34% of women with worse QoL. Independent factors determining high QoL were age (over 45 years compared to below 25 years, OR = 0.17 [0.07–0.46], p<0.001), BMI (high vs low, OR = 0.47 [0.28–0.80], p = 0.005), having children (OR = 0.30 [0.18–0.48], p<0.001), having surgery for endometriosis (OR = 0.55 [0.32–0.94], p = 0.029), and education (high vs low, OR = 2.75 [1.75–4.31], p<0.001)

**Conclusion:**

Cluster analysis identifies homogeneous women phenotypes for QoL with endometriosis. Implementing new methodological approaches improves QoL of endometriosis women and allows appropriate preventive strategies.

## Introduction

Endometriosis is identified as a chronic disorder where cells similar to those lining the interior of the uterus, known for their secretory function, are discovered in locations outside the uterus. These cells respond to the hormonal shifts of the menstrual cycle, leading to persistent inflammation. Symptoms of endometriosis, which overlap with those of various other conditions, prominently include intense pain during menstrual cycles and sexual activity, abdominal discomfort (at times extending to the sacral area), and pain while urinating and undergoing gynecological evaluations [1–3]. Endometriosis is thought to impact 7% to 15% of reproductive-age women, encompassing 30% to 50% of women facing infertility challenges and nearly half of those experiencing chronic pelvic pain syndrome. These figures are speculative due to the potential for endometriosis to be symptom-free, rendering precise prevalence rates elusive [4,5].

Recent investigations have emphasized the extensive influence of endometriosis on various life factors [6]. Considering the symptomatic and complicating nature of endometriosis, it is crucial to address not only the physical symptoms but also the social and psychological ramifications associated with the diagnosis, including the patient’s quality of life (QoL) [7].

Evaluating QoL is crucial to identify the most suitable management and treatment strategies, taking into account the patient’s overall health and their physical, psychological, and social wellness [8]. The WHO conceptualizes QoL as the individual’s perception of their life situation within their cultural and value system context, reflecting their aspirations, standards, and concerns, influenced by their environment. QoL metrics cover the capacity to maintain social roles, adaptability, psychological health, and social interactions.

The significant prevalence of endometriosis, coupled with its social and economic consequences, garners considerable research interest in the QoL domain [6,9]. As QoL is inherently subjective, varying greatly based on numerous personal and external factors, it remains essential to better understand the factors associated with QoL in endometriosis. Moreover, in the context of new challenges in personalized, predictive, and preventive medicine, it is essential to understand the harmful factors which could influence QoL. Thus, creating a phenotype of women with risk of low QoL could be of interest in the personalized medicine which is currently implemented. Cluster analysis is a multivariate methodology that can be performed to identify groups of participants with similar characteristics in the context of complex mechanisms it is a methodology for performing groups in which the data are not scattered evenly by n‐dimensional space but instead form clusters. This approach has been recently performed for several models, including recently for endometriosis [10,11]. Cluster analysis based on clinical variables has been observed to be mainly effective in the exploration of the characterization of phenotypes in diseases. Several findings have suggested that cluster analysis could improve the characterization of a disease phenotype. This novel approach has not yet been applied to QoL in women suffering from endometriosis. Thus, our aim was to investigate the hypothesis that cluster analysis could be used to identify homogeneous phenotyping subgroups of women according to the burden of the endometriosis for their QoL, and then to investigate the phenotype differences observed between these subgroups.

## Methods

### Study design and participants

We designed and conducted a cross-sectional survey using survey software developed by our hospital. The survey was completed anonymously to encourage honest and unbiased responses.

The study link was disseminated via social media (Instagram) where participants were asked to forward this link to others they know. All registrants were free to accept or decline the invitation, with no monetary reward received in return. Participants were also informed that they could withdraw at any time. Following internationally accepted ethical codes, respondents were duly informed of the purpose of the survey and were reminded of their participation rights before proceeding to take the survey. A research protocol was conducted to obtain approval from an ethical committee. The distribution of the questionnaire occurred between November 2023 and January 2024 in France on social media (Instagram). We closed the survey link after the workshop ended.

### Questionnaire and measuring instruments

The questionnaire was developed and adapted based on a review of literature [12–14]. It was pretested among six health and social care professionals and modified according to their feedback.

The survey was conducted in French and required approximately five minutes to complete.

The questionnaire was divided into the following sections (**S1 File**):

- Sociodemographic questions (marital status, age, educational level, children, BMI level calculated as weight (in kg) divided by height squared (in meters) and categorized as high (BMI > 30 kg/m^2^), moderate (BMI between 25 and 30 kg/m^2^), and low (less than 25 kg/m^2^).
- Questions related to the disorder (diagnosis, symptoms, treatment, age at diagnosis etc.)
- Symptoms of endometriosis were defined as: pain during sexual intercourse; abnormal or heavy menstruation; infertility; pain during urination during periods; pain during bowel movements during periods; other digestive issues (diarrhea, constipation, nausea); worsening pain over time; pain, particularly excessive menstrual cramps and other symptoms.
- EHP-5 questionnaire: The EHP-5 (Endometriosis Health Profile) is a tool for measuring health-related quality of life in endometriosis [15]. It is a two-part questionnaire reffering to the last 4 weeks. The first part is a 5-item core questionnaire including questions about pain, control and powerlessness, emotions, social support and self-image. The second part is a 6-item modular questionnaire that consists of questions that may not be applicable to every woman with endometriosis. These 6 items refer to work life, relationship with children, sexual intercourse, medical, treatment and infertility. Each of the 11 items is scored on a Likert-type scale with the range from 0 = never to 4 = always. The second part also has an option ‘not applicable.’ Scores are then transformed on a scale 0–100, with 0 = best possible health status, 100 = worst possible health status.

**Ethics statement.** The study was approved by the Foch IRB: IRB00012437 (approval number: 23-07-05) on 27 July 2023. Written consent was obtained from all participants.

### Statistical analysis

Characteristics of the study population were described as the mean standard deviation (SD) for continuous variables. Categorical variables were described as numbers and proportions. Comparisons between groups were performed using the Mann–Whitney test or t Student test for continuous variables. Pearson’s *χ*2 test was performed for categorical variables. Good QoL was defined as EHP-5 considered inferior to the lower 25^th^ percentile, as there is no cutoff existing in literature to define good QoL based on EHP-5. K‐means, a major clustering algorithm, was performed to show structure in data and divide participants into groups [10,16]. Principal compound analysis, mapping high‐dimension data into low‐dimension space, was performed to diminish the primal data into two dimensions. Here we excluded participants without missing data. The main steps in the K‐means algorithm were: (1) Select initial cluster centers with the number of K, (2) Assign each point to its closest cluster center, and (3) Compute new cluster centers. In step 1, K points are defined randomly as initial cluster centers. In step 2, when we assign each point to its closest cluster center, we compute the distance, such as the Euclidean distance, between points and centers. In step 3, the new cluster centers are computed as the mean of all points belonging to each cluster. The optimal number of clusters showing the best fit was selected using the highest cubic classification criterion (CCC), which estimates the number of clusters using Ward’s minimum variance method. Covariables selected for the construction of the clusters were: pain during sexual intercourse, abnormal or heavy menstruation, infertility, pain during urination during periods, pain during bowel movements during periods, other digestive issues (diarrhea, constipation, nausea), worsening pain over time, pain particularly excessive menstrual cramps and other symptoms.

Then, the two clusters with 100% of good classification of good or low EHP-5 were compared by multiple logistic regression models computing odds ratios (OR) with 95% confidence interval (95% CI) and adjusted for covariables with p value <0.20 in univariable analysis. Statistics were performed using SAS software (version 9.4; SAS Institute, Carry, NC). A *p* value < 0.05 was considered statistically significant.

## Results

1,586 women responded to the questionnaire. The characteristics of the women are shown in **Table 1**. The 25^th^ percentile of EHP-5 was used to define good or low EHP-5, the cutoff was 600 in the dataset.

**Table 1:** Characteristics of the study population according to the different clusters.

	Clusters																	
	1		2		3		4		5		6		7		8			
	N = 65	4.1%	N = 291	18.4%	N = 201	12.7%	N = 72	4.5%	N = 234	14.7%	N = 410	25.9%	N = 44	2.77%	N = 269	16.9%	P value*	P value**
**Age**																	**<0.001**	**0.092**
18–25 years	10	15,38%	13	4,47%	18	8,96%	14	19,44%	44	18,80%	100	24,39%	4	9,09%	34	12,64%		
26–30 years	10	15,38%	55	18,90%	19	9,45%	20	27,78%	72	30,77%	92	22,44%	14	31,82%	40	14,87%		
31–35 years	16	24,62%	71	24,40%	44	21,89%	6	8,33%	50	21,37%	84	20,49%	6	13,64%	50	18,59%		
36–40 years	12	18,46%	92	31,62%	44	21,89%	18	25,00%	40	17,09%	70	17,07%	6	13,64%	53	19,70%		
41–45 years	10	15,38%	36	12,37%	42	20,90%	10	13,89%	16	6,84%	46	11,22%	6	13,64%	60	22,30%		
More than 45 years	7	10,77%	24	8,25%	34	16,92%	4	5,56%	12	5,13%	18	4,39%	8	18,18%	32	11,90%		
**Surgery**	9	13,85%	54	18,56%	24	11,94%	14	19,44%	24	10,26%	62	15,12%	2	4,55%	50	18,59%	0.015	0.076
**Education**																	<0.001	<0.001
Moderate	18	27,69%	72	24,74%	53	26,37%	16	22,22%	60	25,64%	98	23,90%	12	27,27%	58	21,72%		
High	4	6,15%	47	16,15%	32	15,92%	16	22,22%	80	34,19%	56	13,66%	8	18,18%	56	20,97%		
Low	43	66,15%	172	59,11%	116	57,71%	40	55,56%	94	40,17%	256	62,44%	24	54,55%	153	57,30%		
**EHP-5**	606.9	133.7	799.7	111.3	636.7	172.1	656.9	170.6	476.1	99.8	755.4	98.7	556.8	197.7	706.9	132.5	<0.001	<0.001
**EHP-5<25**^**th**^ **percentile**	26	40,00%	8	2,75%	52	25,87%	22	30,56%	234	100,00%	0	0,00%	18	40,91%	28	10,41%	<0.001	<0.001
**BMI (kg/m2)**	26.7	5.4	27.1	6.4	25.9	5.9	24.3	4.5	24.7	5.5	25.5	5.3	25.9	5.4	26.7	6.0	<0.001	<0.001
**BMI level**																	<0.001	0.003
High	17	26,15%	82	28,57%	40	19,90%	10	13,89%	32	13,91%	90	21,95%	10	22,73%	83	30,86%		
Moderate	24	36,92%	83	28,92%	63	31,34%	14	19,44%	50	21,74%	111	27,07%	8	18,18%	50	18,59%		
low	24	36,92%	122	42,51%	98	48,76%	48	66,67%	148	64,35%	209	50,98%	26	59,09%	136	50,56%		
**Having children**	33	50,77%	142	49,48%	106	52,74%	28	38,89%	62	26,72%	208	50,73%	20	45,45%	160	59,48%	<0.001	<0.001
**Being in a couple**	57	87,69%	245	84,19%	160	79,60%	54	75,00%	170	72,65%	322	78,54%	30	68,18%	209	77,70%	0.015	0.093
**Tobacco smoking**	14	22,22%	81	27,84%	32	16,08%	16	22,22%	50	21,37%	129	31,46%	6	14,29%	82	30,71%	<0.001	0.005
**Menopause**	18	27,69%	24	8,30%	20	9,95%	10	13,89%	22	9,40%	50	12,20%	8	18,18%	54	20,07%	<0.001	0.274
**Treatment for endometriosis**	23	35.38%	109	37.46%	72	36.18%	20	27.78%	110	47.01%	195	47.79%	10	22.73%	140	52.04%	<0.001	<0.001
Type of treatment																	0.847	0.173
Surgery	9	42,86%	54	50,47%	24	35,29%	14	70,00%	24	22,64%	62	32,80%	2	20,00%	50	36,23%		
Hormonotherapy	12	57,14%	51	47,66%	36	52,94%	6	30,00%	78	73,58%	122	64,55%	6	60,00%	74	53,62%		
Infertility treatment	0	0,00%	2	1,87%	8	11,76%	0	0,00%	4	3,77%	5	2,65%	2	20,00%	14	10,14%		

*Comparison between all clusters.

**Comparison between clusters 5 and 6.

K‐means clustering was implemented with 8 clusters proving the best fit with the optimal CCC value of 17.2162 according to the different symptoms of endometriosis (**Table 2**). The characteristics of each cluster are shown in **Table 1**.

**Table 2:** Number of K means clusters analyses, and optimal cubic classification criterion (CCC).

Method	No. Clusters	CCC	Best
K Means Cluster	4	-3,1610	
K Means Cluster	5	-3,8399	
K Means Cluster	6	4,38815	
K Means Cluster	7	6,61179	
K Means Cluster	8	17,2162	Optimal CCC
K Means Cluster	9	16,9506	
K Means Cluster	10	15,8145	
K Means Cluster	11	12,9871	
K Means Cluster	12	11,4099	

A cluster was highlighted (cluster 5) showing a 100% rate of good QoL (**Table 3**). This cluster presented only rates of pain during sexual intercourse, pain during bowel movements during periods, other digestive issues and pain, particularly excessive menstrual cramps, that is felt by more than 70%. Another cluster (cluster 6) showed a 100% rate of worse QoL (**Table 3**). It presented rates of pain during sexual intercourse, abnormal or heavy menstruation, pain during bowel movements during periods, other digestive issues, worsening pain over time and pain, particularly excessive menstrual cramps, that is felt by more than 70%.

**Table 3:** Characteristics of the variables used for the construction of the 8 clusters.

Cluster	N	EHP -5<25^th^ percentile	pain during sexual intercourse	abnormal or heavy menstruation	infertility	pain during urination during periods	pain during bowel movements during periods	other digestive issues (diarrhea, constipation, nausea)	worsening pain over time	pain, particularly excessive menstrual cramps that are felt	other symptoms
1	65	40%	100%	27%	24%	9%	23%	0%	66%	15%	53%
2	291	2%	96%	85%	100%	74%	85%	100%	83%	83%	44%
3	201	25%	0%	50%	34%	22%	35%	100%	55%	50%	49%
4	72	30%	91%	77%	33%	69%	75%	0%	58%	72%	13%
5	234	100%	79%	68%	16%	47%	82%	100%	62%	76%	28%
6	410	0%	94%	78%	0%	77%	97%	100%	89%	81%	46%
7	44	40%	0%	31%	27%	9%	22%	0%	50%	45%	36%
8	269	10%	100%	34%	17%	8%	20%	100%	67%	28%	62%

**Fig 1:**
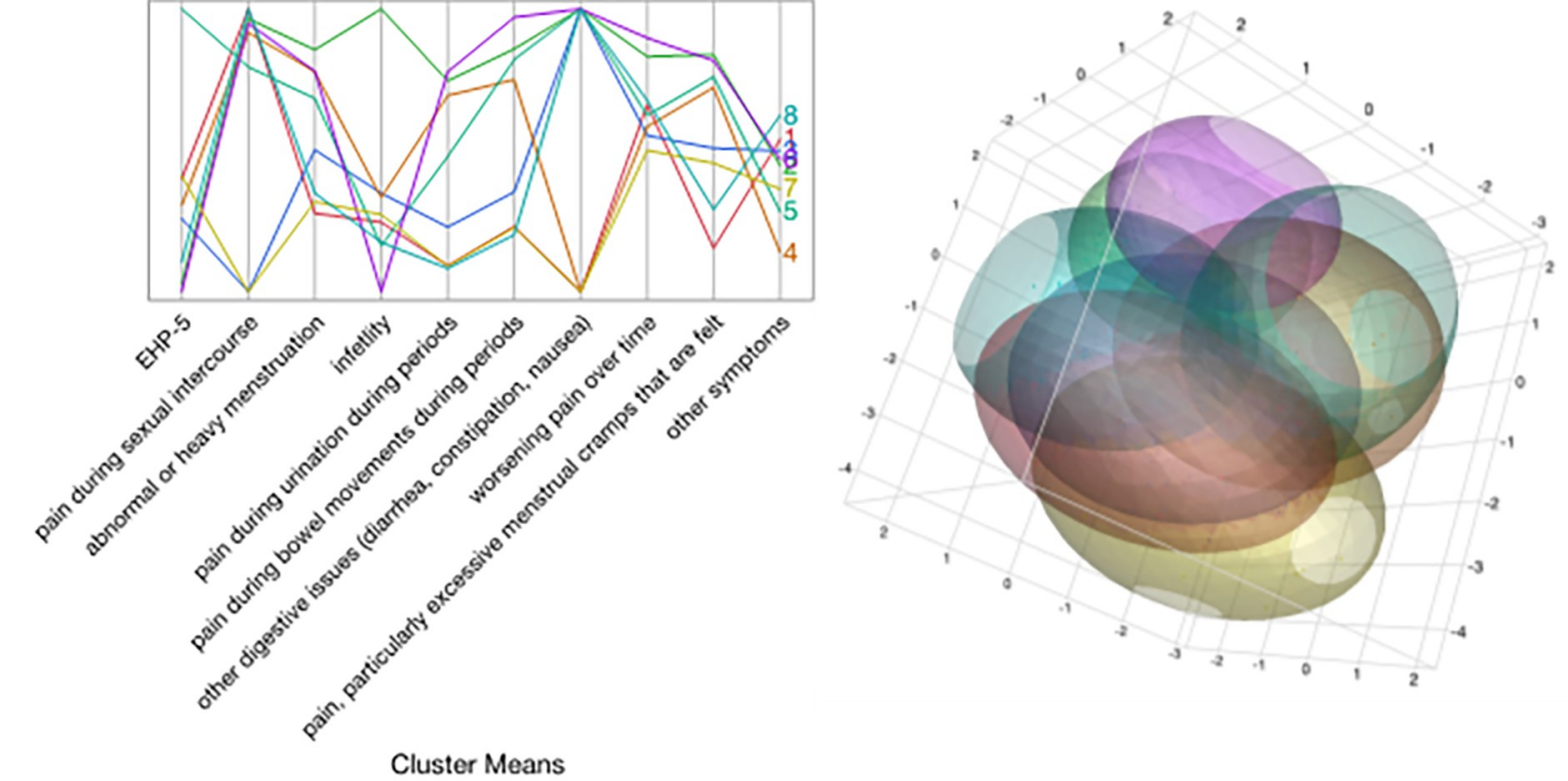
K‐Means clustering method. Parallel coordinate plots for the display of the structure of the observations in each cluster showing how the clusters differ, and biplot 3B of the clusters.

Parallel coordinate plots for the display of the structure of the observations in each cluster show how the clusters differ. This figure presents the different cluster hierarchies **(Fig 1**).

Clusters 6 and 8 with 100% of classified rates represent 644 women for 41% of the population (**Table 3**).

Cluster 5 and 6 were significantly different for all the parameters, except for menopause status (p = 0.274), being in a couple (p = 0.093), and having surgery for endometriosis (p = 0.076) (**Table 1**). After applying a multiple regression logistic, the independent factors determining participants from cluster 8 to the cluster 6 according to QoL were age (over 45 years compared to below 25 years, OR = 0.17 [0.07–0.46], p<0.001), BMI (high vs low, OR = 0.47 [0.28–0.80], p = 0.005), having children (OR = 0.30 [0.18–0.48], p<0.001), having surgery for endometriosis (OR = 0.55 [0.32–0.94], p = 0.029), and education (high vs low, OR = 2.75 [1.75–4.31], p<0.001) (**Table 4**).

**Table 4:** Multiple logistic regression model for QoL between cluster 5 and cluster 6.

Parameters	OR 95% CI	P value
Age		
18–25	Ref.	
26–30	0.41 [0.15–1.11]	0.080
31–35	0.56 [0.23–1.35]	0.198
36–40	0.47 [0.19–1.13]	0.091
41–45	0.37 [0.15–0.92]	0.034
46–50	0.17 [0.07–0.46]	<0.001
Surgery	0.55 [0.32–0.94]	0.029
BMI		
High	0.47 [0.28–0.80]	0.005
Moderate	0.61 [0.40–0.95]	0.028
Low	Ref.	
Having children	0.30 [0.18–0.48]	<0.001
couple	1.18 [0.76–1.82]	0.456
Education		
High	2.75 [1.75–4.31]	<0.001
Moderate	1.45 [0.95–2.25]	0.086
Low	Ref.	
Tobacco smoking	0.68 [0.45–1.03]	0.066

## Discussion

The K‐means cluster analysis presented 8 interested clusters for different profiles of participants. This classification remains experimental due to non-clinical validation in care and the need for validation on another dataset of these clusters. Nevertheless, the K‐means clustering allowed us to discriminate two different clusters of homogeneous participants for QoL. In one cluster, the women presented a high level of QoL and represented 234 women for 60% of women with high level of QoL, and another with 410 women for 34% of women with poor QoL.

Exploring the diversity in QoL necessitates distinguishing specific subgroups within the population that exhibit unique patterns and characteristics. This method enables the delineation of phenotypes within these subgroups, delineates the distribution of disease patterns among them, and paves the way for the development of more personalized approaches to patient care. Utilizing non-hierarchical clustering techniques, such as the K-means algorithm, has proven effective in classifying patients into meaningful groups based on clinical and biological data, thereby pinpointing distinct phenotypes within patient subgroups. The findings from this research pinpointed two distinct patient subgroups, both showcasing a 100% QoL, which collectively represent 40% of the participants in the study.

Clinical practice guidelines could tailor their advice to cater to these two identified patient phenotype subgroups. In dealing with a medically complex patient demographic, it becomes possible to classify these individuals into two unique patient profiles, each suited to distinct approaches in resource distribution and coordinated care plans. This approach yields concrete data that can shed light on complexity models within the general middle-aged population, further acknowledging patient heterogeneity. By pinpointing the critical factors that distinguish the patient profiles within these clusters, this research provides insights to refine patient identification processes and delineate the diverse information necessary for managing complex care prevention effectively.

Among women suffering from endometriosis, age plays a role in determining QoL concerning social connections, with older participants reporting reduced QoL in this area [6]. Moradi et al. uncovered various commonalities and distinctions across different age groups among these patients [7]. Age-independent similarities encompass aspects like marital/sexual relationships, social interactions, and the physical and psychological impacts of the condition. Conversely, disparities were observed in educational opportunities for women younger than 24, employment prospects for those aged 25 to 34, and financial stability for women above the age of 35 [7]. However, this relationship should be considered taking into account the fact that the majority of women were diagnosed around the ages of 35 to 40, and that care management could improve the quality of life despite the aging of the patients [17].

Moreover, women with obesity had the lowest QoL score. This finding is consistent with a previous study showing that underweight women reported a lower QoL than their counterparts of normal weight [8]. Furthermore, some studies have shown that household may negatively impact the QoL of endometriosis women, such as childcare [18,19]. In contrast, the relationship between educational level and QoL remains with inconclusive findings [20,21].

In our study, 74% of women reported pain during sexual intercourse. These women exhibited reduced QoL scores in both psychological aspects and social interactions. The discomfort encountered by women with endometriosis during sexual activities adversely affects their QoL, leading to diminished sexual desire, pelvic pain, and orgasms that are both less frequent and less fulfilling. Consequently, women suffering from endometriosis often feel less relaxed and less content concerning sexual activities [22]. Pain is the major complaint among women living with endometriosis [5]. Several studies have observed that pain has the main negative impact on how women with endometriosis function [23,24], pain is the most significant factor affecting QoL parameters in women living with endometriosis [25].

A possible hypothesis could be central sensitization in women with endometriosis which significantly worsens their quality of life by amplifying pain, even in the absence of active lesions [26]. This heightened pain sensitivity leads to chronic discomfort, impacting physical activities, emotional well-being, and sleep quality. It can also cause sexual dysfunction, strain relationships, and complicate daily functioning. The constant pain contributes to anxiety, depression, and stress, creating a vicious cycle that further diminishes overall life satisfaction. Additionally, traditional treatments may be less effective due to the nervous system involvement, requiring multidisciplinary approaches to improve pain management and quality of life [27].

### Limitations

The limitations of this study include a non-representative French woman with endometriosis. As the link to the questionnaire was made by social media, we are unable to provide the response rate and data collection without information on the localization of respondents. These factors may impact the generalizability of the observed results. Nevertheless, compared to studies focusing on QoL and endometriosis with a similar number of participants [28,29], we can compare our results to the literature due to our 1,586 responders. Our study was internet-based, which introduces the possibility of selectivity bias. Being a cross-sectional study, it does not allow for the establishment of causality. The questionnaire was designed to be simple and easy to answer, limiting our ability to evaluate daily habits. The French ethical guidelines for our anonymous questionnaire prevented us from asking participants about their locations and more detailed personal information. This restriction made it challenging to compare our results with literature focused on these topics.

## Conclusion

Eight clusters were identified with two specific clusters based on the burden of sympotms of endometriosis for the classification of QoL. Independent determinants differencing these two specific clusters of women were age, education, BMI, having children and surgery of endometriosis. Cluster modeling permits the identification of homogeneous women phenotypes for QoL with endometriosis. The implementation of new methodological approaches could be essential to improve the QoL of women living with endometriosis and lead to implementation of appropriate preventive strategies.

## Supporting information

S1 File Word translation of the questionnaire used. (DOCX)
