# Supplementary material for: Quality of life identification by unsupervised cluster analysis: A new approach to modelling the burden of endometriosis
Source: PLoS One. 2025 Jan 16;20(1):e0317178. doi: 10.1371/journal.pone.0317178 (PMC11737779; doi:10.1371/journal.pone.0317178)
Supplement: S1 File — (DOCX) [file pone.0317178.s001.docx]

*Introductory text*

Studies have shown that endometriosis can affect the psychological well-being of patients but also their sexual functioning and social relationships. The quality of sex life is a very important part of overall quality of life. Patients with endometriosis may complain of dyspareunia, which is painful intercourse, impaired overall sexual functioning, and decreased satisfaction, which can negatively impact personal relationships. To date, very few studies in France have been conducted on this subject.

Through this completely anonymous questionnaire, we want to ask you about your quality of life and sexual relationships in order to better understand the repercussions of endometriosis on your overall well-being and to better take care of yourself.

**QUESTIONNAIRE for women with endometriosis**

How old are you? 18-25 / 25-30 / 30-35 / 35-40 / 40-45 / 45-50 / 50-55 / over 55 years old

Do you have children: yes/no

Are you in a relationship/married: yes/no

What is your weight? Listing in kg (from 0 to 100)

What is your size listing in cm (from 0 to 300)

What is your level of education:

- - CAP / BEP
  - Baccalaureate
  - Bac + 3
  - Bac + 5
  - Other

Do you currently smoke: yes/no

At what age were you really diagnosed with endometriosis? Age listing (from 0 to 100)

At what age did you have the first pains suspecting the disease? Listing page (from 0 to 100)

Are you menopausal? (Missed for more than 12 months): yes/no/don't know

Do you have treatment for endometriosis: yes/no

If yes: surgery / hormone therapy / infertility treatment

Do you have (or had) infertility problems related to endometriosis: yes / no / don't know

Frequency of use of painkillers to manage endometriosis: never/only during menstruation/all the time

Pain scale measurement: VAS from 0 to 10 (0 no pain, 10 extreme pain)

How do you rate your quality of life: 0 to 10 (0 very poor quality, 10 very good quality)

Do you have any symptoms related to endometriosis: yes/no

If yes: which ones (multiple choice)

- Pain during sex
- Abnormal or heavy menstruation
- Infertility
- Pain when urinating during menstruation
- Pain during bowel movements during menstruation
- Other digestive problems (diarrhea, constipation, nausea)
- Worsening of pain over time
- Pain, especially excessive menstrual cramps that are experienced.
- Other

All questions are mandatory below

**Questions about your quality of life related to endometriosis**

In the last 4 weeks, how many times, because of your endometriosis:

Did you have difficulty walking because of your pain?

Never

Rarely

Sometimes

Often

Always

Do you feel like your symptoms were regulating your life?

Never

Rarely

Sometimes

Often

Always

Did you have any mood swings?

Never

Rarely

Sometimes

Often

Always

Do you feel like others didn't understand what you were going through?

Never

Rarely

Sometimes

Often

Always

Did you feel like your appearance had changed?

Never

Rarely

Sometimes

Often

Always

Have you been unable to meet professional obligations because of the pain?

Never

Rarely

Sometimes

Often

Always

Did you find it difficult to take care of your child(ren)?

Never

Rarely

Sometimes

Often

Always

Have you felt worried about having sex because of the pain?

Never

Rarely

Sometimes

Often

Always

Did you have the feeling that the doctors thought it was all in your head?

Never

Rarely

Sometimes

Often

Always

Were you disappointed because the treatment didn't work?

Never

Rarely

Sometimes

Often

Always

Did you feel depressed about the possibility of not having children or other children? »

Never

Rarely

Sometimes

Often

Always
